# A critical review of life cycle assessment studies of woody biomass conversion to sugars

**DOI:** 10.1098/rsta.2020.0335

**Published:** 2021-09-20

**Authors:** Niamh Ryan, Polina Yaseneva

**Affiliations:** ^1^ Department of Chemical Engineering and Biotechnology, University of Cambridge, Philippa Fawcett Drive, Cambridge CB3 0AS, UK; ^2^ Cambridge Institute for Sustainability Leadership, University of Cambridge, 1 Trumpington Street, Cambridge CB2 1QA, UK

**Keywords:** life cycle assessment, woody biomass, biotechnology

## Abstract

Woody biomass could potentially become a viable raw material for the future sustainable chemical industry. For this, a suitable regulatory framework must exist, that would create favourable economic conditions for wood biorefineries. Such policies must be developed on the basis of scientific evidence—in this case, data supporting the environmental advantages of the bio-based feedstocks to the chemical industry. The most suitable methodology for comprehensive evaluation of environmental performance of technologies is life cycle assessment (LCA). In this review, the available LCA studies of woody biomass fractionation and conversion to bulk chemical feedstocks are critically evaluated. It has been revealed that the majority of the openly available studies do not contain transparent inventory data and, therefore, cannot be verified or re-used; studies containing inventory data are reported in this review. The lack of inventory data also prevents comparison between studies of the same processes performed with different evaluation methods or using different system boundaries. Recommendations are proposed on how to overcome issues of commercial data sensitivity by using black-box modelling when reporting environmental information. From several comparable LCA studies, it has been concluded that today the most environmentally favourable technology for wood biomass fractionation is organosolv.

This article is part of the theme issue ‘Bio-derived and bioinspired sustainable advanced materials for emerging technologies (part 1)’.

## 1. Introduction

Today's global environmental challenges are demanding industries to decarbonize chemical manufacturing, and scientific communities to develop alternative methods of production of the most important industrial chemicals, without the use of fossil-based feedstocks. A technological success story in bio-renewable chemicals is the production of ethanol from sugars; sugars-based routes to many other molecules in current commercial use have also been developed [1]. However, expansion of the first-generation feedstocks has led to competition with food and attention has shifted to the second-generation bio-feedstocks for the production of bio-based molecules, such as lignocellulosic biomass (grass crops, woody biomass) [2]. Utilization of wood and/or residues of timber and paper industries for the production of low carbon-impact chemicals is particularly attractive in the current environment of the rapidly developing concept of circular bioeconomy [3]. To speed up the adoption of the second-generation bio-based routes to chemicals, it is necessary to show that these routes are both economically viable and environmentally beneficial.

There is an ongoing discussion of the environmental benefits of replacing fossil-based molecules and materials with the bio-based ones. Assessment of environmental impacts is typically done on the basis of life cycle assessment (LCA), the analytical tool covered by the international standards' framework (ISO 14040 and 14044), and the main instrument for comparison of environmental performance of alternative products and processes. However, recent LCA studies have not been able to provide a conclusive answer to the question of whether bio-based molecules offer a lower environmental impact compared to the fossil-based ones [4–6]. The main reasons for this are the differences in the set goals and scope of various LCA studies, and the use of different methodological approaches by LCA practitioners. This results in significant variations of the calculated impact values for the same bio-based chemicals reported in different LCA studies, which are then being compared with the *averaged* LCA data for the fossil-derived chemicals. As LCA results are often reported in the form of the aggregated impacts without presenting explicit process inventory data, it is often impossible to disaggregate the reported information to uncover the sources of differences in the results. While the requirement to report full inventories is stipulated in the LCA standard, this is rarely adhered to.

**Figure 1.**
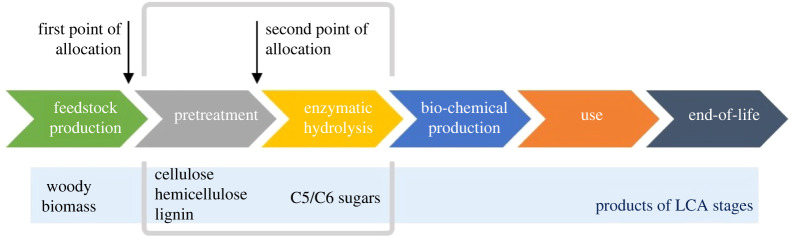
Cradle-to-grave life cycle stages of a bio-chemical. Grey frame represents the boundary of the study. (Online version in colour.)

Generally, a cradle-to-grave type of system boundary of a life cycle model of a bio-based chemical could be split into six stages, as shown in figure 1. Each process step results in certain ecological burdens, and the calculated magnitude of these burdens will depend on: (i) the choices of processes within the stage (choices of feedstock, pretreatment technology, hydrolysis parameters, etc.) and (ii) the allocation methodologies applied to different stages. Between several possible options, the routes via sugars are currently the most economically viable, as they open the largest number of possibilities for the production of different functional end-product molecules [1]. It has been reported that biomass processing steps leading to the production of sugars (pretreatment and enzymatic hydrolysis) constitute one of the major sources of variations in LCA results [6] and, therefore, the focus of this critical review is on technologies of *woody biomass* conversion to sugars and their associated environmental impacts (figure 1). We deliberately left other lignocellulosic feedstocks outside the scope of this study, since consideration of the chemically different feedstocks adds further uncertainty to LCA results. The reviews of LCA studies of other lignocellulosic feedstocks can be found elsewhere [7].

The present review has four objectives: (i) to overview the existing methods of woody feedstocks pretreatment, which have reached at least a pilot or a commercial demonstration scale, (ii) to review the existing LCA studies of bio-chemicals production (via sugars) from woody feedstocks, (iii) to attempt a comparison of the environmental impacts of technically different routes to production of sugars, and (iv) to report the studies where inventory data are available. To the best of our knowledge, it is the first comparison of environmental performance among the different technologies for conversion of woody biomass to sugars.

## 2. An overview of pretreatment technologies for woody biomass

Woody lignocellulosic biomass has a complex polymer structure composed of cellulose, hemicellulose and lignin constituents [8]. The diversity of lignocellulosic feedstocks and variability of composition has posed technological challenges for the development of cost-effective and scalable methods of separation of woody biomass into its components. Nevertheless, a variety of approaches for wood fractionation have been investigated, with some reaching pilot scale or commercial demonstrations. Table 1 gives an overview of technologies specializing in wood fractionation into polysaccharides and lignin in Europe and North America and their scales, excluding studies that are limited to the laboratory scale. Most of these demonstration-scale methods are based on variations of the aqueous acidic biomass pretreatment. There are also a number of alternative technologies currently at pilot- or demo-scales, such as the modified organosolv lignocellulose separation or the supercritical solvent treatment.

**Table 1.** Overview of woody biomass pretreatment technologies existing at pilot or demonstration scale.

technology	company	feedstock	scale	key details of pretreatment process	reference
AVAP®	American Process Inc.	lignocellulosic biomass	demonstration plant built in 2010 with capacity of 1000 t yr^−1^ of biomass	SO_2_–ethanol–water fractionation	[9,10]
BALI™	Borregaard	feedstock agnostic, currently uses Norway spruce	demonstration plant capable of processing 365 t yr^−1^ of biomass	sulfite cooking step	[11]
CelluAPP®	SEKAB	forest residues	demonstration plant in Sweden, capacity 700 t yr^−1^ dry biomass	steam acidic pretreatment (sulfuric acid or SO_2_), enzymatic hydrolysis	[12]
CIMV technology	CIMV	hardwood (and other lignocellulosic biomass)	demonstration plant in France capable of processing 180 kt yr^−1^ of biomass	acid pretreatment using an organic acid	[13–15]
Dawn Technology™	Avantium	forestry and agricultural and residues	pilot plant in the Netherlands, building commercial plant	hydrochloric acid fractionation	[16]
Sunburst™	Sweetwater Energy	hardwood	demonstration plant in Estonia being built	mechanical + dilute acid rapid pretreatment, enzymatic hydrolysis	[17]
Bio-Sep technology	Bio-Sep	forestry and agricultural residues	pilot plant located in UK processing 1500 t yr^−1^ of biomass	ultrasonically enhanced organosolvent fractionation	[18]
Plantrose™ process	Renmatix	feedstock agnostic	demonstration plant in Georgia, USA capable of processing 1000 t yr^−1^ of dry biomass	supercritical water hydrolysis	[19]
TMP-Bio	FPInnovation	hardwood	construction of a biorefinery, capable of processing 100 t yr^−1^ of biomass since May 2019 in Canada	thermomechanical fractionation	[20]

Six out of nine processes described in table 1 are variations of the acidic treatment. The AVAP® process developed by American Process Inc. uses SO_2_–ethanol–water to fractionate lignocellulosic biomass to a high-purity cellulose-rich solid, a hemicellulose solution and lignosulfonates. The presence of sulfur dioxide means the process can be used for fractionation of a mixture of lignocellulosic feedstocks, including softwoods [9,10]. Borregaard's BALI™ process has been implemented in a demonstration plant since 2012, producing lignin and bioethanol [11]. The main pretreatment stage is a sulfite cooking step, whereby most of the lignin is dissolved as lignosulfonate, together with some of the hemicellulose, while cellulose is left mostly intact in the pulp. The ‘spent sulfite liquor’ and the pulp can then be separated, following which the pulp is hydrolysed by enzymes into a sugar chemistry platform comprising monosaccharides. In CelluAPP® technology developed by SEKAB, the forest biomass separation step involves high-temperature steam acidic pretreatment (sulfuric acid or SO_2_), resulting in cellulose, hemicellulose and lignin streams. The technology has a demonstration plant in Sweden, converting 2 t of dry matter per day, and is now being scaled up to 800 t of dry biomass per day [12]. The Compagnie Industrielle de la Matière Végétale (CIMV) pilot plant in France has been operating since 2006, processing lignocellulosic biomass into a variety of products including paper pulp and glucose from cellulose, C5-sugars from hemicellulose and lignin for the chemical industry [13,14]. The process involves treating biomass at atmospheric pressure with a mixture of acetic acid, formic acid and water [15]. The Dawn technology™, developed by Avantium, converts lignocellulosic biomass to high-quality glucose, wood extractives and lignin using concentrated hydrochloric acid at relatively mild temperatures [16]. Avantium opened the Dawn pilot plant in 2018 and the technology is being commercialized within production of the bio-based polymer polyethylene furanoate (commercial plant with capacity of 5 kt yr^−1^ is under construction) [21]. Sweetwater Energy patented Sunburst™ technology, which delivers fast woody biomass pretreatment, where wood is heated for 20 s in the presence of a dilute acid. Since the pretreatment is fast, very few inhibitory chemicals form in the process, yielding materials with unique properties [17]. Currently, the SWEETWOODS project is building a demo-plant based on the conversion of hardwood to sugars and to high-quality lignin [22].

There are other technologies on the cusp of commercialization involving alternative wood separation methods. The Bio-Sep technology combines acid and organosolv biomass pretreatment with an ultrasonically enhanced separation process that does not require high temperatures or pressure. The biomass is mechanically mixed with a dilute organic solution prior to the addition of appropriate amounts of ethanol and methyl isobutyl ketone (MIBK). It is then treated at moderate temperatures with ultrasound prior to centrifugation to separate the liquid and the solid streams, further separation of which yields lignin, hemicellulose and cellulose streams. It is cost-effective at a small enough scale to allow localized processing to take place, but will also be easy to scale up due to its modular nature [18]. Renmatix Inc. developed the Plantrose™ process that produces sugars from a variety of biomass sources including woody biomass by hot water extraction and autohydrolysis of hemicelluloses to C5/C6 sugars, leaving most of the lignin and cellulose intact. The second step uses supercritical water to solubilize cellulose and hydrolyse it to glucose. The lignin contained in the biomass can be recovered as a co-product; it is free from sulfur and easy to isolate from the supercritical hydrolysis residue [19]. In 2019, FPInnovation commissioned a biorefinery to be located in Thunder Bay, Canada, which would be capable of processing 100 metric tons of lignocellulosic biomass per year through a patented thermomechanical treatment process, TMP-Bio [23]. The process separates hardwood biomass particularly well into valuable extractives (antioxidants, flavonoids), hemicellulose, cellulose and lignin [20]. The pilot plant trials have demonstrated that carbohydrate conversions in excess of 90% can be achieved. The process can also be easily retrofitted into an existing mechanical pulp mill [20].

## 3. An overview of life cycle assessment studies of woody biomass conversion to bio-chemicals

In this section, we review LCA studies for the production of chemicals from wood-based feedstocks, and discuss the reported feedstock conversion to sugar technologies, their environmental impacts and availability of life cycle inventory (LCI) datasets. The availability of such datasets is particularly important for transparency and comparability of LCA studies. Environmental impacts calculated on the basis of LCA are often reported normalized to a ‘functional unit’, for example, per kilogram of glucose. When life cycle impacts are reported with respect to a chosen functional unit, but inventories are not provided, it is then impossible to re-calculate to any other functional units which would allow comparison with other studies or use of these data in comparative assessments.

As utilization of second-generation biomass and especially forestry residues is a relatively new concept, the literature focused on environmental assessments is still quite scarce. We have identified 24 studies focused on LCA of processes converting woody feedstocks to bio-chemicals (see table 2). Most of the studies investigated the concepts of biorefinery producing ethanol as the main product; however, a number of authors investigated the environmental impacts of production of upstream chemicals from woody feedstock. We have identified six such studies (see table 2) and we describe them briefly below.

**Table 2.** A list of life cycle assessment studies of woody biomass conversion to bio-chemicals.

authors	biorefinery product	pretreatment method	feedstock	functional unit	inventory data availability
Nuss & Gardner [24]	polyitaconic acid	kraft pulping	softwood	1 kg of polyitaconic acid	
Chen *et al*. [25]	PET bottle	dilute sulfuric acid	Douglas fir	1 kg of PET bottle	✓
Van Uytvanck *et al*. [26]	ethylene glycol	dilute sulfuric acid	willow	500 ml PET bottle	
Aryapratama & Janssen [27]	adipic acid	acid and alkaline	forest residues	1 kg of adipic acid	
Patel *et al*. [28]	polybutelene succinate	SE, organosolv, modified CIMV	willow	1 kg PBS	
Bello *et al*. [29]	HMF, FDCA	dilute sulfuric acid	hardwood chips	1 kg h^−1^ FDCA	✓
Laure *et al*. [30]	glucose, lignin and xylose	organosolv	beech woodchips	50 t of dry wood h^−1^	
Budzinski & Nitzsche [31]	ethylene, organosolv lignin and biogas	organosolv	beech woodchips	400 kt of dry wood yr^−1^	
Bello *et al*. [32]	glucose/hemicellulose/lignin, bioethanol/lignin/furfural	organosolv	beech woodchips	1 t h^−1^ hardwood chips	✓
Li *et al*. [33]	bioethanol	dilute sulfuric and organic acid	forest residues	1 MJ ethanol	
Moncada *et al*. [34]	glucose production	organosolv	spruce woodchips	1 kg of dry glucose	✓ mass/energy flows
Bright & Strømman [35]	bioethanol	dilute sulfuric acid	poplar chips	1000 km distance	
Mu *et al*. [36]	bioethanol	dilute sulfuric acid	poplar woodchips	1 l of ethanol	✓
González-García *et al*. [37]	bioethanol	dilute sulfuric acid	poplar, eucalyptus	1 kg of ethanol	
Olukoya *et al*. [38]	bioethanol	mild bisulfite	redcedar	1 MJ of ethanol	
Liptow *et al*. [39]	ethylene	SO_2_ catalysed pretreatment	sawmill woodchips	50 000 t of ethylene	
Modahl *et al*. [40]	cellulose, lignin, vanillin and bioethanol	Borregaard	spruce woodchips	1 tonne cellulose, lignin powder and vanillin and 1 m^3^ ethanol	
Shadbahr *et al*. [41]	bioethanol	dilute sulfuric acid and steam	poplar woodchips	2 levels: 1 kg of pretreated woodchips and 1 kg of ethanol	
Nwaneshiudu *et al*. [42]	fermentable sugars	mild bisulfite	forest residues	1 kg of dry sugars	
Ganguly *et al*. [43]	iso-paraffinic kerosene	mild bisulfite	softwood residues	1 GJ of iso- parrafinic kerosine	✓
Blanco *et al*. [44]	glucose production	dilute sulfuric acid	forest residues	1 kg of glucose	
Fu *et al*. [45]	bioethanol	steam explosion	fir	1 km distance driven by passenger car	✓ mass/energy flows
Budsberg *et al*. [46]	bioethanol	SO_2_ catalysed steam	willow	1 MJ of ethanol	
Olofsson *et al*. [47]	bioethanol	SO_2_ catalysed steam	spruce	1 MJ of fuel	✓ mass/energy flows

Nuss & Gardner [24] performed attributional LCA of production of polyitaconic acid from softwood biomass to compare the environmental performance of wood-based material with the fossil-based one. In this study, xylene is recovered as a side product from Kraft pulping and the functional unit of 1 kg of dry polymer is used. Mass allocation is applied in xylene extraction and impacts are shown as contributions of the process stages on a graph; however, the unit's conversion is not possible.

Both Chen *et al*. [25] and Van Uytvanck *et al*. [26] looked at the environmental impacts of polyethylene terephthalate (PET) bottle production from various bio-based feedstocks (including softwood residues) versus fossil-based feedstocks. Chen considered multiple scenarios with both PET production reagents—ethylene glycol (EG) and terephthalic acid (TPA)—being produced from a bio-based source (via mild bisulfite pretreatment) and presented a comprehensive LCI based on the data obtained from industrial collaborators. Van Uytvanck *et al*. [26] investigated the environmental impacts of producing a PET bottle using ethylene glycol derived from willow, sugarcane and corn stover. The biomass separation model was based on the National Renewable Energy Laboratory (NREL) design of a dilute acid treatment process [48]. Both studies have found that under certain production scenarios, the bio-based options could be superior to the fossil-based production.

Aryapratama & Janssen [27] compared life cycle impacts of producing adipic acid from forest residues and fossil-based feedstock. Different preatreatment scenarios were considered (acid and alkaline pretreatments with and without energy integration). The overall impacts data were presented graphically only, and broken down to processes contributions; however, for a reader, it is difficult to figure out values of impacts from these graphs and the limited inventory information presented.

Patel *et al.* [28] compared greenhouse gas emissions of polybutylene succinate plastic production from first- (corn) and second-generation (willow) bio-feedstocks with the fossil-based analogues. The study considers steam explosion, organosolv and CIMV organosolv biomass pretreatments. In the life cycle model, biomass pretreatment was aggregated with the production of succinic acid and, therefore, it is impossible for us to decouple the contributions of the pretreatment step towards the impacts.

Bello *et al.* [29] presented an LCA of production of 5-hydroxymethylfurfural (HMF) and 2,5-furandicarboxylic acid (FDCA) from hardwood chips. The functional unit of the LCA was 1 kg h^−1^ of FDCA and a dilute acid pretreatment step was used. However, in this study, biomass separation and hydrolysis are grouped into one step and no inventory details are provided. The impact of the combined forestry/pretreatment/hydrolysis step in all considered impact categories is reported to be negligible, except in the case of agricultural land occupation where this step contributes over 60% of the overall impact.

Another group of studies we reviewed are the studies considering ethanol production from woody feedstock. These represent the bulk of existing literature on conversion of wood to bio-chemicals.

A study comparing environmental performance of bioethanol produced from different lignocellulosic feedstocks was carried out by Falano *et al.* [49]. The biorefinery model was built on the dilute pretreatment model described in NREL reports [50,51]. The authors concluded that among wheat straw, poplar, miscanthus and forest residues, poplar demonstrates better environmental performance in most of the considered impact categories, with forest residues being the second best option. The authors compared their findings with the fossil-based ethanol, but could not conclusively say that bioethanol is environmentally a better option.

We could not identify any studies explicitly comparing the environmental impacts of ethanol production from woody biomass on the basis of different pretreatment methods. However, there are few studies comparing the effect of pretreatment methods for other types of lignocellulosic biomass [52–54]. The biomass pretreatment methods considered in most of the bioethanol studies are variations of acidic pretreatments, but few studies consider organosolv wood biomass pretreatment within the biorefinery production processes.

For example, Laure *et al*. [30], Budzinski & Nitzsche [31] and Bello *et al*. [32] investigated environmental impacts of different biorefinery concepts based on processing beech wood and using organolsolv biomass pretreatment. Laure *et al.* [30] and Bello *et al*. [32] were looking at a biorefinery producing glucose, lignin and xylose. Both authors used the functional unit of amount of biomass treated per hour for the study of emissions of the biorefinery. Laure mass allocated biorefinery emissions for its products, while Bello only presented the breakdown of emissions by relative process contributions (in per cent), making it impossible to compare these two studies. However, Bello *et al*. [32] present life cycle inventories for each process step, which are based on the Aspen Plus model developed by other authors [55].

Budzinski & Nitzsche [31] analysed the environmental performance of four beech wood-based biorefinery concepts with ethylene, organosolv lignin and biogas as the main products. Water–ethanol organosolv was used as the biomass separation method. Numerical values for all environmental impacts were given for biorefineries as a whole, with no analysis of the inputs from different process stages.

Li *et al*. [33] compared the use of low boiling point aprotic solvents, tetrohydrofuran (THF) and acetone, for extracting forest residues biomass sugars in ethanol biorefineries. The biomass pretreatment method considered in this study involves mixing with dilute sulfuric acid and an organic acid. The study uses 1 MJ of ethanol (E100) produced as the functional unit. The only environmental impact presented is the greenhouse gas emissions from the process and for each solvent considered, the data are given in terms of emissions for each input to the process.

Moncada *et al.* [34] compared the environmental impacts of C6 sugars production from spruce chips in biorefineries using organosolv and wet milling separation technologies. The functional unit of the study is 1 kg of dry glucose. Impacts are presented as one value for the overall process with different allocation scenarios (100% allocation to glucose, mass allocation to all products (C6 sugars, furfural, lignin) and for scenarios with and without energy integration).

The next group of the reviewed studies considered the environmental impacts of bioethanol biorefineries with variations within the acidic biomass separation. The majority of these studies are based on simulations using data from NREL reports [50,51,56,57].

Both Bright & Strømman [35] and Mu *et al*. [36] compared the environmental impacts of bioethanol production via gasification and fermentation routes considering poplar woodchips as a feedstock and based their simulations on the same NREL report [57]. Bright also referenced impacts to those of the fossil-based fuel. The studies used different functional units, making comparisons between the calculated impacts difficult. Neither author disaggregated environmental impacts in terms of the contributions of process steps (apart from feedstock production); however, Mu presented brief inventories of impacts per inputs.

González-García *et al*. [37], Budsberg *et al*. [46] and Shadbahr *et al*. [41] used the updated version of the NREL report [50] to calculate impacts of bioethanol production from woodchips. The ethanol production system described in the NREL report is based on the process design and economics of producing ethanol from corn stover. González-García investigated cradle-to-grave life cycle impacts of producing an 85–15%v/v blend of ethanol/gasoline from three different lignocellulosic crops: black locust, eucalyptus and poplar. Shadbahr in their study compared two modified pretreatment methods: the base case simply uses the pretreatment method described in the NREL report, while the second one uses a higher sulfuric acid concentration and a longer residence time in the pretreatment reactor. Budsberg investigated the environmental impacts of ethanol production from willow crop biomass using a sulfur dioxide catalysed steam explosion pretreatment. Although based on similar simulation data, it is impossible to compare these studies due to differences in functional units, assessment methodology applied and lack of life cycle inventories.

Olukoya *et al.* [38] performed LCA of the production of bioethanol from eastern redcedar using a mild bisulfite pretreatment. The process simulation was based on the NREL report on the production of ethanol from corn stover [51]. The impacts presented are greenhouse gas emissions, non-renewable energy use and water usage, with a functional unit of 1 MJ of ethanol produced. The study reports that pretreatment contributed to 65% of GHG emissions.

Liptow *et al.* [39] compared the environmental impacts of producing ethylene from sawmill woodchips via gasification and fermentation routes. Inventory data for the wood fermentation were sourced from Liptow [58] (based on the ‘low enzyme consumption’ scenario described therein), where sulfur dioxide catalysed steam pretreatment was used. Similar to previous studies, ethanol production is considered as a single stage in the evaluated environmental impacts and the chosen functional unit does not allow comparison with other studies.

Modahl *et al.* [40] conducted environmental impact assessment of a biorefinery in Norway based on Borregaard technology, producing cellulose, ethanol and other chemicals from Norwegian spruce. The impacts are presented in relation to the product system (functional unit: 1 t of the product for cellulose, lignin, vanillin and 1 m^3^ for ethanol) and no industrial inventories are given.

Nwaneshiudu *et al.* [42] and Ganguly *et al*. [43] both based their LCAs on the 2011 NREL report [51] but for different process boundaries. Nwaneshiudu investigated the conversion of forest residues to fermentable sugars, whereas Ganguly conducted an LCA of the production of iso-paraffinic kerosene from softwood residues for use as jet fuel. Studies consider different functional units, but Ganguly presents full LCI information for all process stages. However, inventory values are inconsistent and their use in further studies may to lead to erroneous conclusions.

In a recent study, Blanco *et al.* [44] compared life cycle impacts of glucose production from woody biomass residues with production from maize starch. The biomass separation was modelled based on the NREL report [56] and the functional unit in this study is 1 kg of glucose.

Fu *et al*. [45] analysed the potential environmental benefits and limitations of using bioethanol produced from balsam fir as a transport fuel. The pretreatment method used is steam explosion and the functional unit is 1 km distance driven by a passenger car. The process model is based on industrial data and mass and energy flows are provided in the paper.

Olofsson *et al.* [47] investigate environmental impacts of producing bioethanol from wood (spruce) using the sulfur dioxide catalysed steam pretreatment method. The focus of the study is on enzymatic hydrolysis and the impacts are presented per MJ of fuel with regard to on-site and off-site enzymes production. Authors present mass and energy flows inventories for the considered model.

The literature reviewed in this section was analysed in terms of the availability of LCI information (table 2) and comparability of environmental impacts for woody biomass pretreatment/hydrolysis technologies. The results and discussion of this comparison are given in the next section.

## 4. Comparison and discussion of life cycle assessments for different conversion technologies of woody biomass into sugars

Within the scope of this study, we identified and compared life cycle impacts attributed to different pretreatment/hydrolysis technologies discussed in the open literature. As we consider glucose to be the most promising commodity chemical for bio-based chemicals production and kilograms of sugar is one of the most commonly observed functional units between the studies (table 2), 1 kg of sugar was chosen as a functional unit for comparison between different studies.

We identified eight studies where impacts can be assigned to a unified functional unit, three of which used mass of sugar explicitly [34,42,44] and five others that reported data potentially allowing the functional unit conversion [25,30,32,40,43]. However, we could not use three out of these eight studies for comparison due to lack of detail or inconsistency in the presented information.

The comparison has taken in account life cycle impact assessment methods, allocation approaches and availability of energy integration (table 3). As the scope of the paper is to review analysis of data on pretreatment technology and enzymatic hydrolysis step, contribution of the feed production step was excluded where possible (figure 1, study boundary). The motivation behind this was the diversity of possible feedstocks (different types of trees) and allocation methods (feed was considered as pure material (felled wood) or as a waste stream (forest/sawmill residues)). Because of this, conclusions on whether feedstock production greatly contributes to the overall impact of sugar production vary a lot across the literature. Thus, Moncada *et al*. [34] attributed 10% of sugar production impact (greenhouse gas emissions) to the feedstock production (spruce wood), while Bello *et al*. [32] attributed 15% of the impact to the feedstock from beech chips. Interestingly, Nwaneshiudu *et al*. [42], who considered wood residues as a feedstock, observed 27% of impact coming from the biomass preparation. The differences in these values also come from the different modelling approaches—whether transport is included or not. Therefore, we left feedstock production comparison out of the scope of this study.

**Table 3.** Methodological details for reviewed LCA studies on woody biomass conversion to sugars. Presented environmental impacts are calculated per 1 kg of sugars (except Modahl *et al*. [40] where functional unit is 1 kg of cellulose).

author	pretreatment	LCIA	climate change	acidification	eutrophication	human toxicity	allocation^a^	feedstock included	energy integration^b^
Moncada	organosolv	ReCiPe	0.63	—	—	0.0492	mass	no	no
Moncada*	organosolv	ReCiPe	0.17	—	—	0.0235	mass	no	yes
Modahl	Borregaard/kraft pulping	CML	1.16	0.0106	0.00356	—	mass	yes	yes
Nwaneshiudu	mild bisulfite	TRACI	0.43	0.1189	0.00017	—	mass	no	yes
Blanco	dilute acid	ReCiPe	0.82	0.0356	—	0.06	all impact allocated to glucose	no	no
Laure	organosolv	CML	0.73	0.0015	0.00131	—	mass	not clear	no

^a^Mass allocation of impacts to sugar stream produced except for Blanco [44].

^b^Other wood fractionation streams are used for energy production for the process.

*Used to differentiate between energy integration scenarios within one study [34].

Out of all environmental impacts assessed in the chosen studies, only climate change was calculated in every study. Other overlapping impacts include acidification, eutrophication and human toxicity. As the studies used different impact assessment methods (table 3), for some impact categories, it was impossible to make a meaningful comparison of the results. Thus, although four studies presented calculated eutrophication potential, due to the difference in impact methods and the underlying emission calculation models [59], it was possible to compare only two of them.

**Figure 2.**
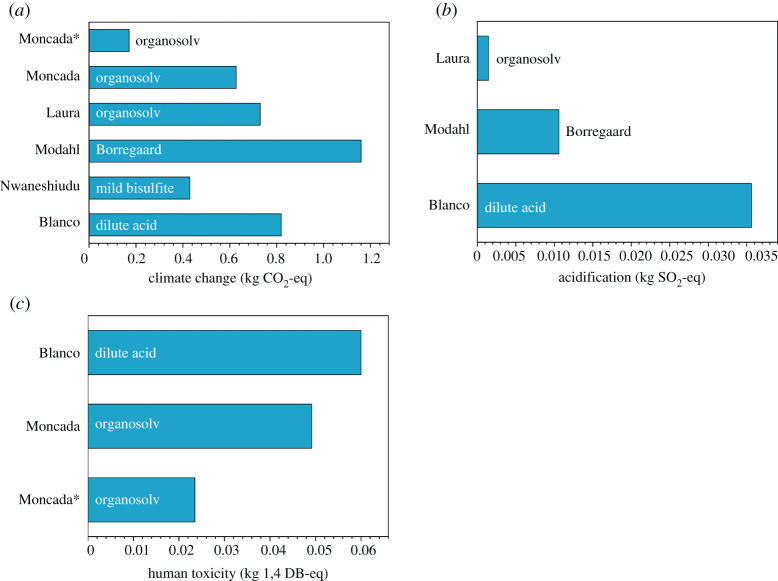
A comparative analysis of (*a*) climate change, (*b*) acidification and (*c*) eutrophication impacts calculated in different studies for wood conversion to sugars (FU of 1 kg of sugars). See table 3 for details. * used to differentiate between energy integration scenarios within one study [34]. (Online version in colour.)

Figure 2 shows climate change, acidification, eutrophication and human toxicity impacts for the analysed studies. The presented impacts refer to the functional unit of 1 kg of sugar for all studies except Modahl *et al*. [40], where impacts are calculated per kilogram of cellulose (meaning that impact values in this case are underestimated in comparison to other studies). As justified above, feed production impacts are excluded from comparison where possible. All of the impacts used for comparison were retrieved from the models using the same allocation method (mass allocation).

We were able to compare processes based on organosolv, dilute acid, mild bisulfite pretreatments and the Borregaard process (figure 2*a* and table 3). Among these, the organosolv-based process with energy integration demonstrated the lowest greenhouse gas emissions (Moncada *et al*. [34]). The same process without energy integration showed very similar impact value to the organosolv-based process investigated by Laure [30] (no energy integration, ethanol recycled). Modahl reported significantly higher climate change impact for cellulose production in the Borregaard process; however, as it was impossible to disaggregate feedstock production from the impact, as the value might be overestimated compared to other studies. Authors considering dilute acid [44] and the mild bisulfite [42] methods reported climate change values differing almost twofold. The reason for this could be the different allocation methodologies, as Blanco assigned the maximum of impact to the glucose production. Only few studies separately reported contribution of the enzymatic hydrolysis stage to the overall climate change impact of sugar production and those reported varied significantly. For example, Blanco reported negligible contribution of enzymatic hydrolysis compared to the pretreatment stage, while Nwaneshiudu assigned 30% of climate change impact to the hydrolysis. A very general conclusion can be drawn from this comparison: energy integration allows us to reduce climate change impact significantly and the organosolv-based process of glucose production seems to create the lowest climate change impact.

We compared acidification between three studies based on organosolv biomass separation, dilute acid separation and the Borregaard process (figure 2*b*). Understandably, the organosolv-based process demonstrated significantly lower acidification impact compared with the Borregaard and the dilute acid-based processes due to the impacts arising from production of reagents in the last two. The Borregaard process study reported considerably lower acidification impact than the dilute acid one.

Human toxicity values could be extracted from two studies only: from the Moncada's organosolv-based study and from the Blanco's dilute acid-based study. Moncada's scenario with energy integration demonstrated the lowest human toxicity impact (figure 2*c*).

We argue that as the quality of LCA studies massively depends on accessibility and quality of the underlying data, and as a lot of LCA data have been generated in different studies, it is important for LCA practitioners to communicate the full inventory data behind their models in a transparent way. It is the only way for other LCA practitioners, whether they are intending to use published data in their models or to verify their own models through meaningful comparisons, to be able to make use of the existing data.

In this review exercise, we reveal that from over 20 LCA studies on conversion of woody feedstocks to bio-based chemicals, only eight studies contained inventories of some form, mainly schemes with mass and energy balances (table 1), and only five presented data in a way that harmonized comparison of wood processing methods could be done. We were able to identify only two LCA studies explicitly mentioning the existing industrial or pilot-scale pretreatment processes (Borregaard [40] and CIMV [28]); however, neither presented inventory data. In reality, this confirms that the majority of LCA data and inventories are not publicly available, significantly hindering progress on decision-making in the transition to more sustainable technologies.

## 5. Conclusion

Woody biomass represents a potentially sustainable source of feedstocks for the manufacture of bio-chemicals from all components of the raw material (cellulose, hemicellulose, lignin and secondary metabolites). However, since separation of wood into components is a complex process, it is not obvious that the overall environmental impacts of a given bio-chemical manufacturing route would be low. Hence, to reach an unequivocal conclusion about environmental advantages of the wood-based biorefineries, it is necessary to obtain reliable and verifiable LCA results.

This review with examples of environmental performance of wood to sugar conversion has demonstrated how from a relatively large number of scientific studies reporting LCA results in peer-reviewed publications, only a few studies can be harmonized in a way that could be used for independent verification or for further use of the data. This highlights the methodological problem—lack of transparent reporting of raw inventory data. Another issue revealed is the lack of consistent LCA results for processes that have already reached high technology readiness levels—at least a pilot-scale or an industrial demonstration scale. However, the studies where inventory data are present and potentially available for reuse are highlighted together with methodological details.

While it is frequently argued that revealing full inventories is impossible due to issues of commercial sensitivity, since a full inventory may be reconstructed to reveal technological details that may form the basis of a competitive advantage, the lack of inventories is hampering transition to sustainable technologies, as decisions on policies to take technologies forward rely on science-based evidence. It is, however, possible even today to represent processes as input–output black-box models, such that any commercially sensitive details are not made public while the important environmental data over the whole supply chain are available. This issue is discussed within the context of full digitalization of the chemistry value chain, which is likely to be reflected in forthcoming national and regional policies. Therefore, we strongly recommend that the approaches described above should be used as guidelines for companies reporting environmental information of their processes.

Nevertheless, this review of the existing LCA studies and technical papers describing woody biomass pretreatment technologies suggests that, currently, organosolv is the most environmentally favourable technology. The second conclusion is that environmental efficiency of the biomass treatment technologies is improved through process integration and careful utilization of all available energy and material resources.

## Data accessibility

This article has no data.
